# Economic burden of multiple sclerosis: a cross-sectional study in Iran

**DOI:** 10.1186/s13561-021-00350-y

**Published:** 2022-01-03

**Authors:** Mehdi Rezaee, Khosro Keshavarz, Sadegh Izadi, Abdosaleh Jafari, Ramin Ravangard

**Affiliations:** 1 Student Research Committee, School of Management and Medical Information Sciences, Shiraz University of Medical Sciences, Shiraz, Iran; 2 Department of Health Management, Policy and Economics, Faculty of Management and Medical Information Sciences, Kerman University of Medical Sciences, Kerman, Iran; 3 Department of Health Services Management, School of Management and Medical Information Sciences, Shiraz University of Medical Sciences, Almas Building, Alley 29, Qasrodasht Avenue, 71336-54361 Shiraz, Iran; 4 Clinical Neurology Research Center, Medical School, Shiraz University of Medical Sciences, Shiraz, Iran

**Keywords:** Economic burden, Multiple sclerosis (MS), Direct medical costs, Direct non-medical costs, Indirect costs

## Abstract

**Background:**

Multiple Sclerosis (MS) is a chronic debilitating disease that imposes a heavy socioeconomic burden on societies. This study aimed to determine the economic burden of MS on patients using the first (CinnoVex and ReciGen) and second (Fingolimod and Natalizumab) drug therapy lines.

**Methods:**

This cost of illness study was an economic evaluation carried out as cross-sectional research in 2019 in southern Iran. A total of 259 patients were enrolled in two lines of drug therapy (178 patients in the first line and 81 ones in the second). The prevalence-based approach and the bottom-up approach were used to collect cost information and to calculate the costs from the societal perspective, respectively. The human capital approach was applied to calculate indirect costs. To collect the required data a researcher-made data collection form was utilized. The data were obtained using the information available in the patients’ medical records and insurance invoices as well as their self-reports or that of their companions.

**Results:**

The results showed that the annual costs of MS in the first and second lines of drug therapy per patient were $ 1919 and $ 4082 purchasing power parity (PPP), respectively, and in total, $ 2721 PPP in 2019. The highest mean costs in both lines were those of direct medical costs, of which purchasing the main medicines in both lines accounted for the highest.

**Conclusion:**

Considering the findings of this study and in order to reduce the burden of the disease, the following suggestions are presented: providing necessary facilities for the production of MS drugs in the country; proper and equitable distribution of neurologists; expanding the provision of home care services; and using the technologies related to the Internet, including WhatsApp, to follow up the MS patients’ treatment.

## Introduction

Multiple sclerosis (MS) is a chronic debilitating disease that leads to severe disabilities [1]. It is an immune-mediated disease that lacks the presence of a specific antigen that drives the inflammatory process [2]. The highest prevalence of this disease is among people of 20–50 years old [3]. In 2016, there were 2,221,188 people with MS worldwide [4]. The prevalence of the disease is increasing in both developed and developing countries, the latter had a low prevalence rate in the past [5, 6]. The results of a study show that the aged-based standardized incidence rate of MS in the world has increased by 10.40% compared to 1990. It has also been stated that the prevalence of the disease has been varied in different countries so that the highest prevalence rates per 100,000 people have been found in North America (*n* = 164.60), Western Europe (*n* = 127), and Australia (*n* = 91.10), and the lowest have been found in Eastern Sub-Saharan Africa (*n* = 3.30), Central Sub-Saharan Africa (*n* = 2.80), and Oceania (n = 2). Furthermore, 18,932 people died due to MS worldwide in 2016 [4].

According to some research, Iran is also classified among countries with moderate prevalence, with an increase in the prevalence of the disease in recent years (7–9). The results of a meta-analysis in 2020 demonstrated that during 1985–2018, the prevalence of the disease in Iran had increased by 2.30% annually [10]. Also, studies conducted in the country represented that the following provinces had the highest prevalence rates of MS: Tehran (*n* = 148.06 per 100,000 in 2018) [11], Isfahan (*n* = 85.80 per 100,000 in 2014) [12], and East Azerbaijan (*n* = 73.26 per 100,000 in 2014) [13]. Besides, the results of a study conducted in Fars province in 2015 showed that this province had high prevalence and incidence rates among the provinces of the country, the former was 72.10 and the latter was 5.20 per 100,000 [14].

MS can have a significant negative impact on the patients’ quality of life [15]. Recurrences in the disease, which are clinically defined as the occurrence of an important neurological event, may lead to hospitalization or be associated with some kind of disabilities that disrupt work as well as social and family life [16, 17]. Furthermore, the disease, which often occurs in the patient’s most productive years of work, can impose great potential costs and heavy socioeconomic burdens on the society [18].

Today, the use of FDA-approved drugs for the treatment of MS has made it possible to better control the disease and adapt the patients to the conditions. The drugs are classified into first-line (Interferon beta-1a (CinnoVex and ReciGen) and Glatiramer acetate) and second-line (including oral Dimethyl fumarate, Teriflunomide, and Fingolimod, as well as Natalizumab and Alemtuzumab) drug therapies [19. The results of some studies and clinical procedures have shown that first-line drug therapies are safer and their most common side effects include injection site reactions, gastrointestinal disorders, and fatigue. However, it has been proven by the safety results of pivotal and post-marketing trials that second-line drugs might also raise safety concerns about the risk of cardiovascular as well as serious infectious diseases [20–22]. There are also hepatic complications in second-line treatments. The results of several clinical trials have indicated that Fingolimod can cause atrioventricular block and slow heart rate. In addition, Alemtuzumab can cause infection-related symptoms (infections such as mucosal herpes and mucosal fungal infections) and cancer [23–26]. In general, despite the more serious side effects of second-line drugs, one of their main advantages is their greater effectiveness and easier use, which result in more patient adaptation to the condition compared to treatment with first-line drugs [27, 28].

However, despite the benefits mentioned above, second-line drugs impose higher treatment costs than first-line ones. In 2004, there was no drug treatment for MS costing less than $ 50,000 a year in the United States, and Natalizumab cost twice as much as Interferons and Glatiramer acetate. With the introduction of Fingolimod to the list of FDA-approved drugs at an annual cost of $ 50,775 in 2010, which was 25% higher than Interferons and Glatiramer acetate, the need for careful control of drug costs increased [28].

Along with the arrival of Natalizumab in Iran in 2004, a circular on the classification of MS drugs into first- and second-line drugs was prepared by the Ministry of Health and Medical Education and provided to specialists to consider when prescribing MS drugs, according to which, specialists prescribe the drugs listed in each drug therapy line.

So far, various studies have been conducted to determine the costs of MS in different countries. For example, Ernstsson et al. (2016) carried out a review study and found that drugs accounted for the main cost of MS in patients with low disease severity, and various studies showed that 29 to 82% of the total cost was that of drugs. However, the main cost components for the patients with more advanced MS symptoms were lost production due to the disease and informal care, accounting for 17 to 67% of the patients’ cost according to various studies [29]. In his study conducted in the United States, Owens (2016) concluded that medication therapy accounted for about 75% of the total cost and increased with the patients’ increasing disabilities [30]. In a study conducted in Poland, Matschay et al. (2008) found that the total direct and indirect costs of MS per patient were € 11,507.15 ($ 7867.07 PPP, adjusted for PPP and inflation) (42.73% of total costs) and € 15,423.81($ 10,544.77 PPP, adjusted for PPP and inflation) (57.27% of total costs), respectively, of which € 9914.44 ($ 6778.19 PPP, adjusted for PPP and inflation [31]) per patient (36.81% of total costs) were associated to medication therapy [32].

Prescott et al. (2004) conducted a study in the United States and found that the mean cost of the disease was $ 12,879 ($ 17,066 PPP, adjusted for PPP and inflation [31]) of which 64.84% was related to medication therapy, 26.24% was the cost of receiving outpatient services, 7.79% was the cost of inpatient services, and 1.13% was that of emergency visits [33].

In Iran, some studies have been carried out on the costs of MS. Rezaei et al. (2019) in their study concluded that direct medical costs accounted for about 78.30% of the costs of the disease, of which about 69.57% was the cost of drug purchase [34]. Another study conducted by Ravangard et al. (2018) indicated that direct medical costs of MS patients accounted for about 51.01% of the total costs, and drug purchase accounted for about 56.76% of direct medical costs as well (35).

Given that most MS patients are of working age, and due to the high prevalence of this disease in Iran and the world, and also due to the high economic costs of the disease, it can be concluded that MS imposes high costs to families and societies. Therefore, calculating the economic burden of MS in Iran and the world and providing the results of such studies to health policymakers and managers to use in planning and allocating sufficient funds to provide services to MS patients is of great importance. On the other hand, to the best of our knowledge, no comprehensive study has been conducted on the measurement of the economic burden of MS using either of the two drug therapy lines in Iran. Thus, this study was conducted on the MS patients referring to the medical centers affiliated to Shiraz University of Medical Sciences in southern Iran in 2019 in order to determine the economic burden of the disease on the patients using the first (CinnoVex and ReciGen) and second (Fingolimod and Natalizumab) drug therapy lines.

## Material and methods

This study was a cost of illness (COI) study carried out as cross-sectional research on the patients with MS in Fars province, who referred to the medical centers affiliated to Shiraz University of Medical Sciences in southern Iran in 2019. According to the statistics by the Special Diseases Center of Shiraz University of Medical Sciences and the Fars MS Society, the number of patients who took ReciGen and CinnoVex to treat their disease was 590 and 650, respectively. Thus, regarding the results of the pilot study and using the following sample size formula, and considering α = 5%, *δ*^2^ = 1.2, and d = 0.25, the sample size for this study was determined to be 89 in each group of patients. The samples were selected using the simple random sampling method based on the random numbers table and were entered into the study.

$$ \boldsymbol{n}=\frac{{\left({\boldsymbol{Z}}_{\mathbf{1}-\boldsymbol{\alpha} /\mathbf{2}}\right)}^{\mathbf{2}}{\boldsymbol{\delta}}^{\mathbf{2}}}{{\boldsymbol{d}}^{\mathbf{2}}} $$

Also, all the patients taking Fingolimod (*n* = 50) and Natalizumab (*n* = 31) who were willing to participate in the study were selected through the census method. The inclusion criteria were the use of the afore-mentioned drugs for at least one year and the willingness to participate in the study. This study was conducted from the societal perspective to identify direct medical costs (DMCs) and direct non-medical costs (DNMCs) as well as indirect costs (ICs). To collect the cost information, the prevalence-based approach was applied, which estimates the direct and indirect costs of all cases generated in a given year for each disease or group of diseases (36). Furthermore, the bottom-up approach was employed to calculate the costs. In the bottom-up method, the resources utilized by each individual are measured and therefore, this method is able to reveal the differences in treatment of the patients [37].

In order to increase the accuracy of the data collected on DMCs, the patients’ inpatient and outpatient medical records and insurance invoices were used. To estimate the costs, the patients were divided into two groups including those who were taking first-line and second-line drugs, respectively. The first-line drugs in this study included Interferon beta-1a for muscle injection under the brand name CinnoVex, and Interferon beta-1a for subcutaneous injection under the brand name ReciGen, and the second-line drugs included oral Fingolimod and injectable Natalizumab.

To obtain an accurate estimate of the costs imposed on the patients with MS using each drug therapy line, the total direct costs of main and supplementary medicines (without subsidies), Physicians’ visits, laboratory tests, Magnetic Resonance Imaging (MRI), physiotherapy and other services, and hospitalization & surgeries in 2019 were separately determined for the patients in each drug therapy line.

The data on DNMCs were obtained from the interviews with the patients. Given that a large percentage of the patients referred to the medical centers were living outside the city of Shiraz, items such as the cost of transportation to the health centers to receive medical services, accommodation costs, and meal costs were considered as the components of DNMCs for the patients and their companions.

The human capital approach was used to calculate the ICs [38]. The ICs per patient were calculated based on the mean daily productivity lost due to absence from work and sick leave days for hospitalization or treatment follow-up, and the mean daily productivity lost for each patient’s companion due to absence from work to accompany or care for the patient. In the present study, the individuals’ wages were utilized to calculate the productivity lost. In addition, the daily wage determined by the Ministry of Cooperatives, Labour, and Social Welfare ($22.9 PPP per day in 2019) was applied as the mean daily wage for housewives and students aged 15–65 years [39]. The ICs data were collected through face-to-face or telephone interviews with the patients who had received inpatient and outpatient services from the intended medical centers in the study year or their companions.

It should be noted that in the present study, all the costs were converted into PPP dollars in 2019 using the PPP exchange rate of each dollar equal to 22,075 Rials [40].

Furthermore, to estimate the number of MS patients, the data on the prevalence of the disease were required by different drug therapy lines in the country, but due to the fact that such information was not available by separate drug therapy lines, the published data related to the prevalence of the disease in the country were first applied to estimate the total number of MS patients in the country and then, according to the information obtained from the Special Diseases Center of Shiraz University of Medical Sciences and also based on the opinion of the neurologists, the percentage of the patients using each drug therapy line was estimated.

According to the studies published in Iran from 2013 to 2018, the mean prevalence of MS in the country was 87.89 per 100,000 people [11–14, 41–44]. Therefore, considering the population of Iran in 2019 (83.27 million people) [45], the total number of MS patients in the country was estimated at 73,186. Moreover, according to the information obtained from the Special Diseases Center of Shiraz University of Medical Sciences, 70% of the patients were using the first line of drug therapy and 30% were using the second. Thus, 51,230 and 21,956 patients were using the first and second lines of drug therapy, respectively.

Finally, after collecting the data on the prevalence rate of the disease, the population of the country, and the mean cost per patient in each line of drug therapy, the economic burden of MS in the country was calculated using the following formula:

Economic Burden: Total cost per patient (DMCs + DNMCs + ICs) × estimated number of MS patients in Iran.

### Sensitivity analysis

In this study, two one-way sensitivity analyses were performed to estimate the costs. In the first analysis, cost components were assumed constant, and the number of patients was changed according to the prevalence rates extracted from various studies. Given that various studies had estimated the prevalence rates of MS in the country at 50.4 to 148.06 people per 100,000 during 2013–2018 [11–14, 41–44], these rates were used as the lower and upper limits of the number of patients, respectively (with a 95% confidence interval). In the second sensitivity analysis, the lower and upper limits of the costs (with a 95% confidence interval) were tested taking into account Iran’s inflation rate data in 2018 and 2019. To this end, according to the inflation rates in 2018 and 2019 in Iran (31.2 and 41.2%, respectively) [46], the inflation rate in 2018 was deducted from the costs obtained and added to the inflation rate in 2019 to obtain the lower and upper limits of the costs. Then, using the information obtained from the one-way sensitivity analyses, a two-way sensitivity analysis was performed (best- and worst-case scenarios) to test the combined effects of varying both the number of subjects and the treatment costs, in which, the mean value of the least cost information represented the best scenario and the mean value of the greatest cost information represented the worst [47].

### Ethical statement

This study was approved by the Shiraz University of Medical Sciences Ethics Committee (Code: IR.SUMS.REC.1395.S275). To participate in this study, all the patients or their companions were taken written informed consent, and all were assured of the confidentiality of their responses.

## Results

In this study, 259 patients were enrolled in two drug therapy lines (178 patients in the first line and 81 ones in the second). Table 1 shows the patients’ demographic characteristics based on the drug therapy lines.

**Table 1:** Demographic characteristics of patients studied in 2019 (*N* = 259)

Demographic characteristics		First line		Second line		Total	
		Frequency	%	Frequency	%	Frequency	%
Gender	Male	32	17.98	15	18.52	47	18.15
	Female	146	82.02	66	81.48	212	81.85
Age	Under 25 years old	21	11.80	3	3.70	24	9.27
	25–34 years old	70	39.33	37	45.68	107	41.31
	35–44 years old	60	33.71	30	37.04	90	34.75
	45–54 years old	26	14.61	9	11.11	35	13.51
	Over 55 years old	1	0.56	2	2.47	3	1.16
Occupation	Employee	22	12.36	6	7.41	28	10.81
	Self-employed	32	17.98	9	11.11	41	15.83
	Student	11	6.18	8	9.88	19	7.34
	Retired	2	1.12	2	2.47	4	1.54
	Housewife	105	58.99	46	56.79	151	58.30
	Unemployed	6	3.37	10	12.35	16	6.18
Type of basic insurance coverage	Social Security	116	65.17	39	48.15	155	59.85
	Iran Health Insurance	59	33.15	36	44.44	95	36.68
	Armed Forces	2	1.12	2	2.47	4	1.54
	Other insurances	1	0.56	4	4.94	5	1.93
	No insurance coverage	0	0.00	0	0.00	0	0.00
Having supplementary insurance	Yes	38	21.35	12	14.81	50	19.31
	No	140	78.65	69	85.19	209	80.69
Education degree	Below Diploma	43	24.16	17	20.99	60	23.17
	Diploma	62	34.83	30	37.04	92	35.52
	Academic education	73	41.01	34	41.97	107	41.31
Place of residence	Local (of Shiraz)	72	40.45	29	35.80	101	39.00
	Non-local	106	59.55	52	64.20	158	61.00

The results demonstrated that in both first and second drug therapy lines, the majority of patients studied were female (82.02 and 81.48%, respectively), in the age group of 25–34 years (39.33 and 45.68%, respectively), housewives (58.99 and 56.79%), covered by Social Security insurance (65.17 and 48.15%, respectively), without supplementary insurance (78.65 and 85.19%, respectively), with academic education (41.01 and 41.97%, respectively), and non-local (59.55 and 64.20%, respectively) (Table 1).

The data on DMCs, DNMCs, and ICs by the first and second drug therapy lines are shown in Table 2.

**Table 2:** Mean annual DMCs, DNMCs, and ICs per patient based on drug therapy lines studied in 2019 ($ PPP)

Studied Lines	First line		Second line		Total	
Costs	Mean costs	%	Mean costs	%	Mean costs	%
**DMCs**						
Physicians’ Visits	124.23	13.95	163.38	5.62	141.52	8.78
Main Medicines	427.63	48.01	1469.74	50.55	798.94	49.58
Supplementary Drugs	34.78	3.90	532.45	18.31	206.87	12.84
Laboratory Tests	67.52	7.58	155.09	5.33	99.70	6.19
MRI	211.90	23.79	184.44	6.34	209.01	12.97
Physiotherapy & Other Services Costs*	24.72	2.77	13.42	0.46	21.60	1.34
Hospitalization & Surgeries	0.00	0.00	389.25	13.39	133.76	8.30
Total	890.79	46.42	2907.76	71.24	1611.40	59.21
**DNMCs**						
Transportation	442.08	63.67	540.12	72.12	489.42	66.63
Accommodation	151.87	21.88	70.16	9.37	128.49	17.49
Meals	100.33	14.45	70.16	9.37	93.06	12.67
Purchasing Auxiliary Tools	0.00	0.00	68.45	9.14	23.52	3.20
Total	694.28	36.18	748.90	18.35	734.49	26.99
**ICs**						
Patient’s absence from work	190.05	56.92	310.48	73.03	237.30	63.19
Patient’s companion’s absence from work	143.81	43.08	115	26.97	138.24	36.81
Total	333.86	17.40	425.15	10.41	375.54	13.80
**Total Costs**	**1918.93**	**100**	**4081.81**	**100**	**2721.43**	**100**

*Electrocardiography, sonography, and radiotherapy

According to Table 2, the mean DMCs of the patients using the first- and second-line drug therapy were $ 890.79 PPP and $ 2907.76 PPP, respectively (46.42 and 71.24% of the total costs). Thus, the DMCs of the patients using the second-line were higher than those of the patients using the first-line. In this cost group, purchasing the main medicines accounted for the highest DMC (first line: $ 427.63 PPP (48.01%); and second line: $ 1469.74 PPP (50.55%)). As observed, the cost of purchasing the main medicine in the second line of drug therapy was higher than in the first line.

In addition, the lowest DMCs of the patients in both lines of drug therapy were those of physiotherapy and other costs ($ 24.72 PPP (2.77%) in the first line due to the lack of hospitalization and surgery costs, and $13.42 PPP (0.46%) in the second).

Also, DNMCs of the patients using the first- and second-line drug therapy were $ 694.28 PPP and $ 748.90 PPP, respectively (36.18 and 18.35% of the total costs, respectively), with DNMCs higher in the second-line group. In this cost group, the cost of transportation accounted for the highest DNMCs in both lines of drug therapy ($ 442.08 PPP in the first line (63.67%) and $ 540.12 PPP (72.12%) in the second). Thus, the cost of transportation in the second line of drug therapy was slightly higher than in the first.

Furthermore, the lowest DNMCs of the patients in the first line of drug therapy were respectively the cost of meals ($ 100.33 PPP (14.45%)), because of no need to buy auxiliary tools and the cost of purchasing auxiliary tools in the second line of drug therapy ($ 68.45 PPP (9.14%)). In addition, the ICs of the patients using the first and second lines of drug therapy were $ 333.86 PPP and $ 425.15 PPP, respectively (17.40 and 10.41% of total costs, respectively). In this cost group, the patients’ productivity lost due to absenteeism was the highest ICs in both lines ($ 190.05 PPP in the first line (56.92%) and $ 310.48 PPP in the second (73.03%)). According to this finding, the productivity lost in the second line was higher than in the first one.

In general, DMCs and ICs of the patients studied in both lines of drug therapy accounted for the highest and lowest health care costs, respectively. This was true for all the patients regardless of their drug therapy lines (DMCs of $ 1611.40 PPP (59.21%) and ICs of $ 375.54 PPP (13.80%), respectively) (Table 2).

Considering the number of MS patients in the country estimated by using the prevalence rate, and regarding the mean costs extracted from the results of this study, the estimated economic burden on all the patients with MS in the country is presented in Table 3. Thus, the mean cost of MS patients in Iran in 2019 was $199,170,584 PPP, of which 52.31% was related to the first drug therapy line and 47.69% to the second. The results also represented that DMCs accounted for the greatest part of the total economic burden of MS in the first and second lines of drug therapy in the country (46.42 and 71.24%, respectively, and in total, 59.21% of the total costs).

**Table 3:** Estimation of total annual costs of MS patients in the country by drug therapy lines in 2019 ($ PPP)

Drug Therapy Lines	Number of Patients in Iran	DMCs		DNMCs		ICs		COI	
		Mean	%	Mean	%	Mean	%	Mean	%
First line	51,230	45,635,352	46.42	35,568,105	36.18	17,103,715	17.40	98,307,172	52.31
Second line	21,956	63,842,200	71.24	16,442,699	18.35	9,334,509	10.42	89,619,408	47.69
Total	73,186	117,931,925	59.21	53,754,387	26.99	27,484,272	13.80	199,170,584	100

Table 4 shows the results of the sensitivity analyses for direct medical, direct non-medical, indirect, and total costs of the MS drug therapy lines studied. In Table 4-A, the number of patients in the country based on different prevalence rates was assumed constant, and cost components were assumed inconstant. In Table 4-B, the number of patients was considered as constant but the cost components changed based on the inflation rate. Finally, in Table 4-C, the number of patients changed with different prevalence rates, and the cost components also changed based on the inflation rate. In this table, the lowest and highest numbers of MS patients were multiplied by the lowest and highest costs and were considered as the best and worst scenarios.

**Table 4:** Sensitivity analyses for DMCs, DNMCs, ICs, and total costs of MS patients based on drug therapy lines studied in Iran in 2019

Disease Type	Number of Patients in Iran	DMC	DNMC	IC	COI
A: One-way Sensitivity analysis ($ PPP)					
First line	29,378–86,303	26,169,322-76,877,576	20,396,319-59,918,234	9,808,024-28,813,017	56,373,665 -165,608,827
Second line	12,590–36,987	36,609,931-107,548,937	9,428,969-27,699,466	5,352,819-15,724,967	51,391,719-150,973,370
Total	41,968–123,290	62,779,253-184,426,513	29,825,288-87,617,700	15,160,843-44,537,984	107,765,384-316,582,198
B: One-way Sensitivity analysis ($ PPP)					
First line	51,230	31,397,122-64,437,117	24,470,856-50,222,164	11,767,356-24,150,446	67,635,334-138,809,726
Second line	21,956	43,923,433-90,145,186	11,312,577-23,217,091	6,422,142-13,180,326	61,658,152-126,542,604
Total	73,186	81,137,165-166,519,878	36,983,018-75,901,195	18,909,179-38,807,791	137,029,362-281,228,865
C: Two-way Sensitivity analysis ($ PPP) best-case scenario and worst-case scenario					
First line	29,378–86,303	18,004,494- 108,551,138	14,032,667- 84,604,546	6,747,921- 40,683,980	38,785,082- 233,839,664
Second line	12,590–36,987	25,187,633- 151,859,099	6,487,130- 39,111,646	3,682,739- 22,203,654	35,357,502- 213,174,399
Total	41,968–123,290	46,527,627- 280,520,346	21,207,693- 127,863,590	10,843,357- 65,375,829	78,578,676- 473,759,765

## Discussion

The management of MS has changed dramatically in recent years, and the associated costs have increased sharply (48). The discovery of second-line drugs over the past few years has made the treatment of the disease more complex and expensive. Although the drugs are very effective, they are associated with significant costs (30). The present study was conducted on the patients referring to the medical centers affiliated to Shiraz University of Medical Sciences in southern Iran in 2019 in order to determine the economic burden of the disease on the patients using the first (CinnoVex and ReciGen) and second (Fingolimod and Natalizumab) drug therapy lines.

The results of the present study demonstrated that the economic burden of MS in the first and second lines of drug therapy and in total, was $98,307,172 PPP (38,785,082-233,839,664), $89,619,408 PPP (35,357,502-213,174,399), and $199,170,584 PPP (78,578,676-473,759,765), respectively. The results of the study by Chen et al. (2017) in the United States, which estimated the economic burden of MS at $ 198 million [49], are consistent with those of the present research. Palmer et al. (2013) in Australia also concluded that the economic burden of MS was $ 1.0883 billion [50], which is inconsistent with the results of the present study, one of the reasons for which is the high ICs of the disease in that country, which accounted for about 50% of the total costs.

The present study also indicated that DMCs had the largest share of total treatment costs of the patients, accounting for 46.42, 71.24, and 59.21% of the total costs of the disease in the first and second line of drug therapy and in total, respectively. Thus, DMCs were the most important cost component for patients with MS. In addition, the results showed that the largest share of DMCs of the MS patients was that of purchasing the main medicines (48.01 and 50.55% in the first and second lines of drug therapy, respectively, and 49.58% of the total DMCs), which could be due to the high price of such drugs in Iran. The results of the present study are consistent with those of the studies by Alowayesh et al. (2019) in Kuwait [51], Imani et al. (2012) in Iran [52], and Matschay et al. (2008) in Poland [32]. In their systematic review, Naci et al. (2010) investigated 29 studies on the costs of MS and represented that the costs of the disease increased significantly with increased disabilities of the patients. Besides, DMCs were among the most important cost components of MS patients with low disabilities, and increased disabilities led to an increase in ICs [53]. Taylor et al. (2006) conducted a study on MS patients in Australia and indicated that DMCs accounted for about 57.48% of the costs, of which 46.37% was related to drug purchases [54]. In a study carried out on MS patients in Spain, Kobelt et al. (2006) found that DMCs accounted for 36.29% of the total costs, of which 52.08% was the cost of purchasing the drugs [55].

However, Schreiber-Katz et al. (2014) in Germany and McCrone et al. (2008) in the United Kingdom concluded in their studies that DMCs and the cost of purchasing drugs accounted for the lowest percentage of costs [56, 57], which is not consistent with the results of the present study, the reasons for which could be the lower price of the drugs as well as the low cost of outpatient care including the costs of visits and laboratory tests, and the low cost of MRI in those countries.

In addition, in this study, DNMCs accounted for 36.18, 18.35, and 26.99% of the total costs of the disease in the first and second lines of drug therapy and in total, respectively, and transportation costs accounted for the largest share of the total DNMCs (63.67, 72.12, and 66.63%, respectively) in the first and second lines. This might be due to the need for frequent visits to specialists to receive services. Furthermore, as most of the patients referred to the medical centers to receive services in both lines of drug therapy were non-local and had to travel to the capital of the province to receive the services, the cost of transportation was the highest. The results of this study are consistent with those of the studies by Schreiber-Katz et al. (2014) in Germany and Kobelt et al. (2006) in the United Kingdom, in which DNMCs accounted for the largest percentage of the total costs, although transportation costs had a small share [56, 58]. The reason for such a discrepancy could be the extent to which auxiliary tools were available to adapt to the environmental conditions at home and work, and their prices in those countries.

Matschay et al. (2008) studied 120 patients in Poland and found that DNMCs accounted for less than 1 % of the total costs, and therefore, had the lowest share of the MS patients’ costs [32].

This inconsistency could be due to the difference in the number of non-local patients in the studies as well as the difference in transportation and accommodation costs in the countries studied.

Moreover, the results of the present study indicated that the ICs had the smallest share of the total treatment costs, accounting for 17.40, 10.41, and 13.80% of the total costs in the first and second lines of drug therapy and in total, respectively. In this cost group, the patients’ productivity lost due to absence from work were the highest in the first and second lines of drug therapy and total (56.92, 73.03, and 63.19% of the total ICs, respectively), which could be due to the reason that most of the patients had to be absent from work and travel to the provincial capital in order to receive treatment. It is noteworthy that the majority of the patients had visited the medical centers without any companions. The results of this study are consistent with those of the study by Imani et al. (2012) [52].

But the results of the studies by Schreiber-Katz et al. (2014) in Germany [56], Matschay et al. (2008) in Poland [32], Taylor et al. (2006) in Australia [54], and Kobelt et al. in Switzerland (2006), the Netherlands (2006), and Germany (2001 & 2006) [59–62] represented that ICs accounted for a relatively high percentage of the total costs (36–45%). This is not consistent with the results of the present study, some reasons for which could be the methods used in these studies as well as the high daily wages of the patients in those countries.

### Study limitations

One limitation of the present study was the self-reporting of the patients or their companions on DNMCs and ICs because they were likely to forget or approximate some of the costs. In addition, intangible costs were not calculated in this study due to the inability to measure them accurately. Finally, because of the small sample size studied in this research, it is necessary to be cautious in using and generalizing the results of the present study.

## Conclusions

In general, due to the high prevalence of MS in Iran and the chronic nature of the disease and the need for lifelong treatment, the costs of the disease treatment can impose a heavy economic burden on the health care system, the insurance system, and the patients themselves. According to the results of the present study, the costs of the patients consuming the second line of drug therapy was higher than that of the patients consuming the first line, and the highest mean costs in both lines of drug therapy were related to DMCs, of which purchasing the main medicines in both lines was the most costly. Regarding DNMCs and ICs, the transportation cost and the patients’ productivity lost due to absence from work accounted for the greatest costs in both lines of drug therapy, respectively.

Considering the obtained results and in order to reduce the economic burden of MS, due to the high share of drug purchase costs and high prices of foreign drugs, it is suggested to provide the necessary facilities for the production of these drugs in the country. In addition, given that the transportation cost accounted for the highest percentage of DNMCs, it is suggested to reduce these costs by proper and equitable distribution of neurologists, expanding home care services for MS patients, and using Internet-based technologies and cyberspace, including WhatsApp, to follow up the patients’ treatment in order to prevent unnecessary travels of the patients.

## Data Availability

The datasets used and/or analyzed during the current study are available from the corresponding author on reasonable request.
